# Deep tissue optoacoustic monitoring of photothermal treatments in the NIR-II assisted with silica-coated gold nanorods

**DOI:** 10.1038/s44303-025-00134-7

**Published:** 2026-01-28

**Authors:** Eva Remlova, Alexander Jessernig, Marcus Bammel, Daniil Nozdriukhin, Yi Chen, Oscar Cipolato, Xosé Luís Deán-Ben, Inge K. Herrmann, Daniel Razansky

**Affiliations:** 1 Institute for Biomedical Engineering and Institute of Pharmacology and Toxicology, Faculty of Medicine, University of Zurich, Zurich, Switzerland; 2 Institute for Biomedical Engineering, Department of Information Technology and Electrical Engineering, ETH Zurich, Zurich, Switzerland; 3 Institute of Energy and Process Engineering, Department of Mechanical and Process Engineering, ETH Zurich, Zurich, Switzerland; 4 Nanomaterials in Health Laboratory, Department Materials Meet Life, Swiss Federal Laboratories for Materials Science and Technology (EMPA), St. Gallen, Switzerland; 5 Ingenuity Lab, Balgrist University Hospital, Zurich, Switzerland; 6 Faculty of Medicine, University of Zurich, Zurich, Switzerland

**Keywords:** Cancer, Materials science, Nanoscience and technology, Optics and photonics

## Abstract

Gold nanoparticles (AuNPs) absorbing light in the near-infrared (NIR) range offer unparalleled benefits for both optoacoustic (OA) imaging and photothermal therapy (PTT), stemming from their ability to transform optical energy into heat. These unique theranostic capabilities are further complemented by the high sensitivity of OA signals to temperature variations. However, AuNPs typically experience rapid photodegradation when exposed to high laser intensities, which hinders their efficient monitoring with OA. To address this critical limitation, we synthesized silica-coated gold nanorods (AuNRs) featuring enhanced photostability and an absorption peak in the second NIR window (NIR-II, 1064 nm) for optimal tissue penetration. Their comprehensive evaluation under exposure to nanosecond-pulsed and continuous-wave (CW) radiation revealed that the synthesized AuNRs are photostable under laser energy densities required for efficient therapy under OA imaging guidance, which was confirmed with electron microscopy images. Real-time volumetric OA mapping of PTT-induced temperature variations was verified using simultaneous thermal camera readings, whilst *post-mortem* experiments in mice corroborated the viability of this theranostic approach in deep biological tissues.

## Introduction

Gold nanoparticles (AuNPs) stand out for their unique ability to convert light into heat. The underlying mechanism driving this phenomenon is the surface plasmon resonance (SPR) effect, which can be finely tuned by adjusting the particle size and shape so that they absorb light for the wavelength range of interest^1–4^. AuNPs featuring absorption peaks in the near-infrared (NIR) are generally preferred in biomedicine due to the enhanced tissue penetration in this spectrum^5,6^. The biocompatibility, ease of synthesis, and functionalization flexibility of AuNPs have further fostered multiple diagnostic and therapeutic applications^7^. Particularly, the dual function of AuNPs as imaging contrast materials and photothermal therapeutic agents positions them as powerful tools in advancing cancer treatments by facilitating real-time monitoring of localized thermal ablation of tumors^8^.

AuNP-assisted photothermal therapy (PTT) holds great promise for selective cancer therapy or as an adjuvant approach to other treatments such as chemotherapy or immunotherapy^9^. PTT can be employed to directly ablate tumors (>50°C)^10,11^ or to induce sensitization to other therapeutic agents (39-45°C)^12^. AuNPs are also widely used as contrast materials in biomedical imaging research. Particularly, they offer unique benefits as optoacoustic (OA) contrast agents due to their spectrally-distinctive and exceptionally prominent per-particle absorption^13–15^. OA further exhibits outstanding sensitivity to temperature changes associated with heat-induced variations in the Grüneisen parameter^16^. The technique could then potentially provide a dual function to image biodistribution and targeting with AuNPs while also monitoring the temperature rise induced by laser exposure in real time.

Despite the apparent synergies between OA and PTT, the effectiveness of OA monitoring of AuNP-mediated PTT is hindered by the strong photodegradation of AuNPs when exposed to high laser intensities. Photothermally-driven melting and reshaping effects have been reported for different types of AuNPs in their pristine form, even for laser fluence values of a few mJ/cm^2^ well below safe laser exposure limits for human skin^17,18^. As an example of exposures at 800 nm, Fales et al. reported 10% and 50% damage thresholds at approximately 14 mJ/cm² and 23 mJ/cm², respectively, for 800 nm resonant nanorods using nanosecond pulses. Importantly, these thresholds fall below the ANSI maximum permissible exposure limit (31.7 mJ/cm² at 800 nm), indicating that degradation can emerge even under “tissue-safe” conditions^19^. The degradation process is rapid and often appears within the first few laser pulses. It is also size-dependent, with larger nanorods exhibiting lower stability. As described previously, nanoparticle deformation improves from below 2 to above 6 mJ/cm when the effective radius of nanoparticles is reduced from 22 to 5 nm^20^. Nanoparticle melting can occur at substantially lower temperatures than the bulk melting point of the metal and further depends on the laser pulse duration^21^. The reduction in absorption associated with AuNP photodegradation not only impacts OA temperature quantification but also reduces the PTT performance. Notably, a higher temperature rise was observed during exposure of AuNPs to a continuous wave (CW) laser relative to a nanosecond-duration pulsed laser with the same output power^22^.

Recent efforts have focused on enhancing the photothermal stability of AuNPs through surface modifications. One particularly effective strategy consists of coating AuNPs with a silica shell. In such, silica-coated gold nanorods have produced up to three-fold higher signals than their uncoated counterparts with the same optical density, demonstrating the effectiveness of the coating as an OA signal amplifier^23–25^. More importantly, silica coating has been shown to prevent photodegradation and consequent OA signal decay for different AuNP shapes. This makes them more effective for imaging and therapy, ensuring consistent photothermal performance for successful OA-guided PTT^26^. However, the performance of such an approach has never been investigated in detail.

This study comprehensively investigates the performance of real-time OA monitoring of PTT assisted with self-developed silica-coated gold nanorods (AuNRs) that were specifically designed to exhibit peak absorption in the second NIR window (NIR-II, 1064 nm) for optimal tissue penetration. Unlike prior studies that evaluated AuNP photostability under isolated conditions, we present a systematic assessment of their degradation behavior under combined CW and nanosecond-pulsed NIR-II laser exposure, enabling deep-tissue penetration. We closely reflect clinically relevant conditions to demonstrate the feasibility of integrated OA-guided PTT. The synthesized AuNRs are shown to manifest enhanced photostability under optical energy densities required for achieving PTT with a CW laser and contrast-enhanced OA imaging with a short-pulsed laser. Accurate and reliable OA temperature mapping is shown, which allows for real-time feedback and improves the precision of thermal ablation during PTT. Through a combination of in vitro and *post-mortem* experiments, including scanning and transmission electron microscopy (SEM and TEM) for morphological analysis, thermal imaging for temperature quantification, and murine tumor hyperthermia for assessing practical situations, we establish the potential of silica-coated AuNRs as a robust platform for image-guided PTT.

## Results

### Enhanced photostability of silica-coated gold nanorods

**Fig. 1:**
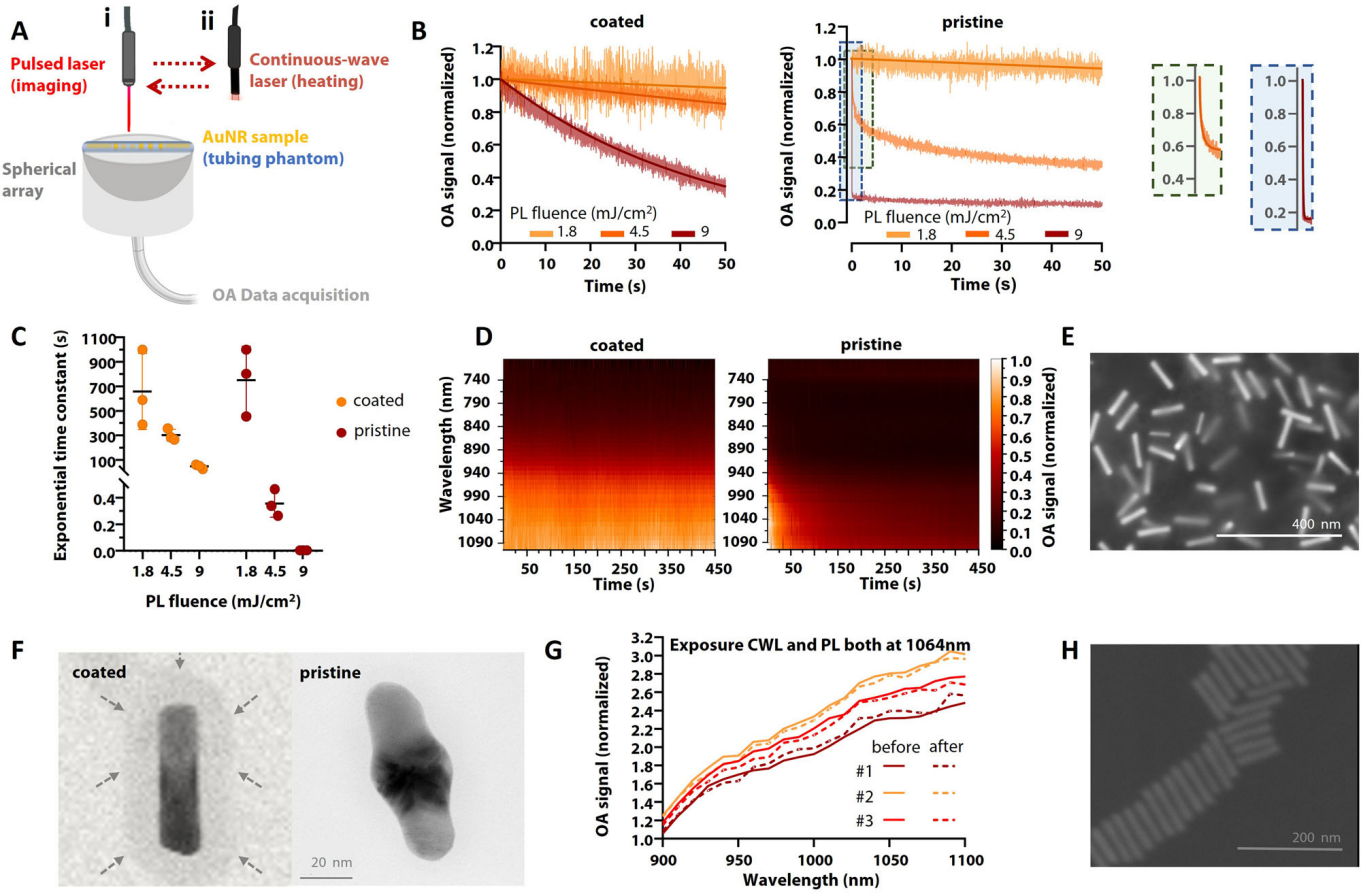
Photostability. **A** Setup consisting of pulsed and CW lasers along with OA array with the sample placed atop and injected into a tubing; **B** OA signal decay for pulsed laser exposure at 1064 nm for three different optical fluence levels; **C** Comparison of photodegradation rates of coated and pristine AuNRs under pulsed laser exposure; **D** Multi-spectral exposure of both coated and pristine AuNRs and their respective photostability and photodegradation in time; **E** SEM image of silica-coated AuNRs under pulsed laser exposure (4.5 mJ/cm^2^); **F** TEM images of silica-coated AuNR under pulsed laser exposure (4.5 mJ/cm^2^) (silica-shell denoted with arrows) (left), pristine AuNR showcasing morphological photodegradation (right); **G** Relative photostability of silica-coated AuNRs before and after simultaneous CW laser exposure (at 8 W/cm^2^) and pulsed laser exposure (at 1.8 mJ/cm^2^), both at 1064 nm for three trials; H) SEM image of pristine AuNRs photostability under CW laser exposure (at 8 W/cm^2^). The figure was partly created using BioRender.

The photostability of silica-coated and pristine AuNRs for laser energy levels required for imaging and therapy was evaluated by exposing the particles to CW and short-pulsed (nanosecond duration) laser beams, as depicted in Fig. 1A. The OA signal decay was monitored for AuNRs exposed to a pulsed laser at 1064 nm with varying fluence (per-pulse energy density) levels, revealing a significant reduction in OA signal intensity for pristine AuNRs compared to their silica-coated counterparts (Fig. 1B). The photodegradation rates of pristine AuNRs were markedly high for fluence levels above 4 mJ/cm^2^, well below safety exposure limits^27^, while silica coating clearly contributed to a significant reduction of decay rates even at higher fluence levels (Fig. 1C). Photodegradation of pristine AuNRs was also observed during multi-spectral (multi-wavelength) laser exposure required for distinguishing (unmixing) the particles from blood and other tissues contributing to the OA background. A superior photostability of silica-coated AuNRs over pristine AuNRs was also clear in this case, with the former maintaining OA signal intensity over time (Fig. 1D). High-resolution SEM images corroborated that no distortion of the AuNR cores was produced following short-pulsed laser exposure (Fig. 1E). TEM images of individual particles further provide a clearer verification of the role of the silica shell in photostabilizing the particles. Silica-coated AuNRs remained intact post-exposure (Fig. 1F, left), whereas pristine AuNRs exhibited substantial morphological changes indicative of photodegradation (Fig. 1F, right).

The photostability of silica-coated AuNRs was additionally evaluated for CW exposure at 1064 nm and power levels required for efficient heating. The particles were imaged with OA before and after CW excitation for 3 minutes, i.e., they were also exposed to a short-pulsed laser for a short time. However, the fluence levels used for OA imaging were within the range where no photodegradation was observed in the measurements described above. Furthermore, no OA signal degradation was observed between trials (Fig. 1G). Note that the second measurement time point was taken 10 minutes after stopping the CW laser so that the sample cooled down to room temperature. Furthermore, SEM images of pristine AuNRs showed no significant morphological instability under these laser parameters (Fig. 1H), ultimately confirming that the pulsed laser is the primary source of photodegradation. This observation is consistent with prior reports showing that nanosecond pulses induce thermal confinement, rapidly heating AuNRs beyond their melting threshold before heat can dissipate, leading to irreversible reshaping, whereas CW irradiation allows gradual thermal dissipation that prevents such morphological changes^1,28^. Importantly, this study not only confirms this mechanism under clinically relevant imaging and therapy conditions, but also provides direct morphological and OA evidence of this degradation pathway. Overall, these findings highlight the photostability of silica-coated AuNRs under higher pulsed laser energies required for OA imaging during PTT.

### Optoacoustic monitoring of temperature

**Fig. 2:**
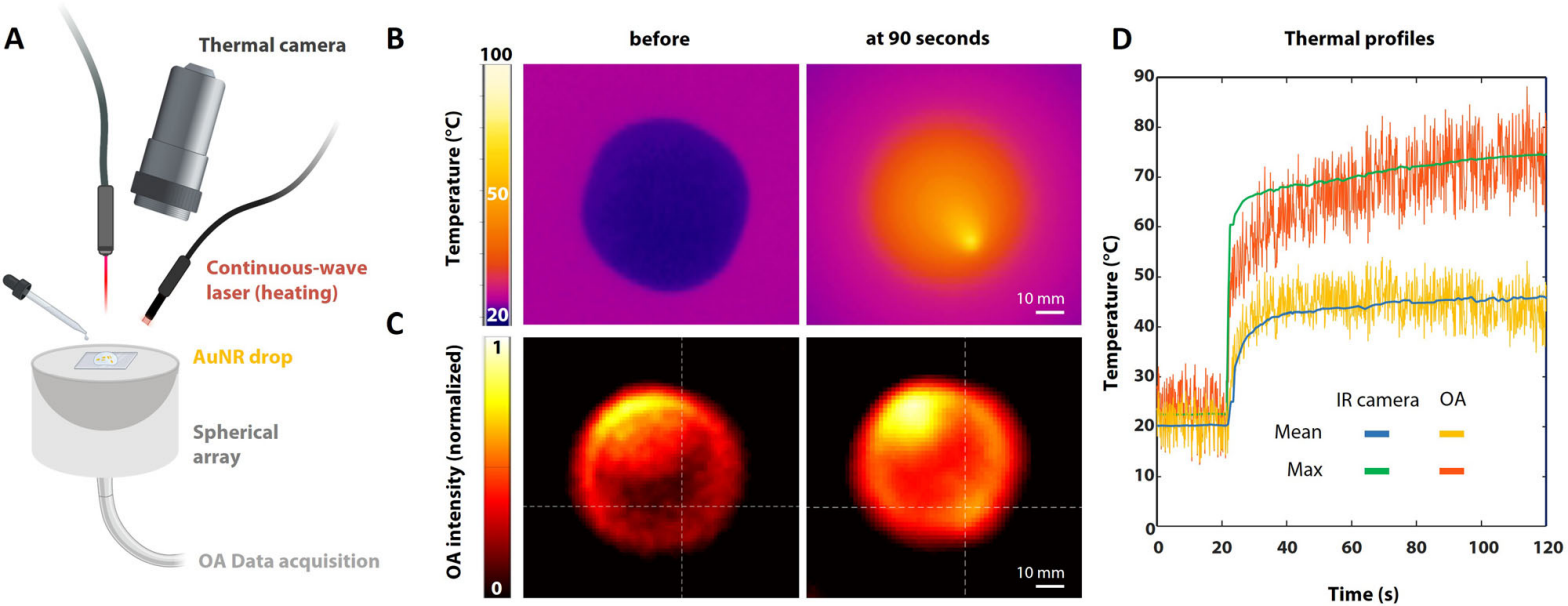
Temperature Estimation with OA. **A** Setup consisting of pulsed and CW lasers along with a mounted IR camera, drop of AuNRs placed atop of OA array on a glass slide; **B** IR camera capturing the baseline sample temperature and heating at 90 seconds; **C** OA image of the baseline and heating at 90 seconds; **D** Thermal profiles of acquired IR and OA data, including the respective mean and max values for each over 2 minutes of heating. The figure was partly created using BioRender.

The feasibility of OA temperature monitoring with the synthesized silica-coated AuNRs during photothermal treatment was assessed using simultaneous OA imaging and reference infrared (IR) thermal camera readings. While OA monitoring renders a 3D view of the temperature distribution at depth, IR thermometry is limited to surface temperature readings. Accordingly, calibration was performed under controlled heating conditions with the sample placement carefully matched. The experimental setup involved a combined pulsed and CW laser system integrated with an IR camera, with a drop of AuNRs placed on a glass slide atop the spherical array transducer used for the OA signal detection (Fig. 2A). Baseline temperature and heating dynamics at 90 seconds were captured by the IR camera, showing a clear increase in thermal emission during the laser-induced heating process (Fig. 2B). OA images acquired at baseline and after 90 seconds of heating provide a means to estimate the temperature distribution. Note, however, that temperature is related to relative OA signal increases rather than absolute signal intensity, the latter being associated to the distribution/concentration of AuNR (Fig. 2C). Temperature increase in the heated AuNR sample was estimated from the OA images based on the known theoretical dependence of Grüneisen parameter on temperature^16,29,30^ (see Methods for details). Quantitative analysis of thermal profiles extracted from both IR and OA data demonstrated strong agreement in mean and maximum temperature values over a 2-minute heating period (Fig. 2D). These results validate the ability of OA imaging to accurately monitor real-time temperature variations, corroborating its potential for precise temperature mapping during AuNR-assisted PTT.

### Optoacoustic monitoring of photothermal therapy in mice

**Fig. 3:**
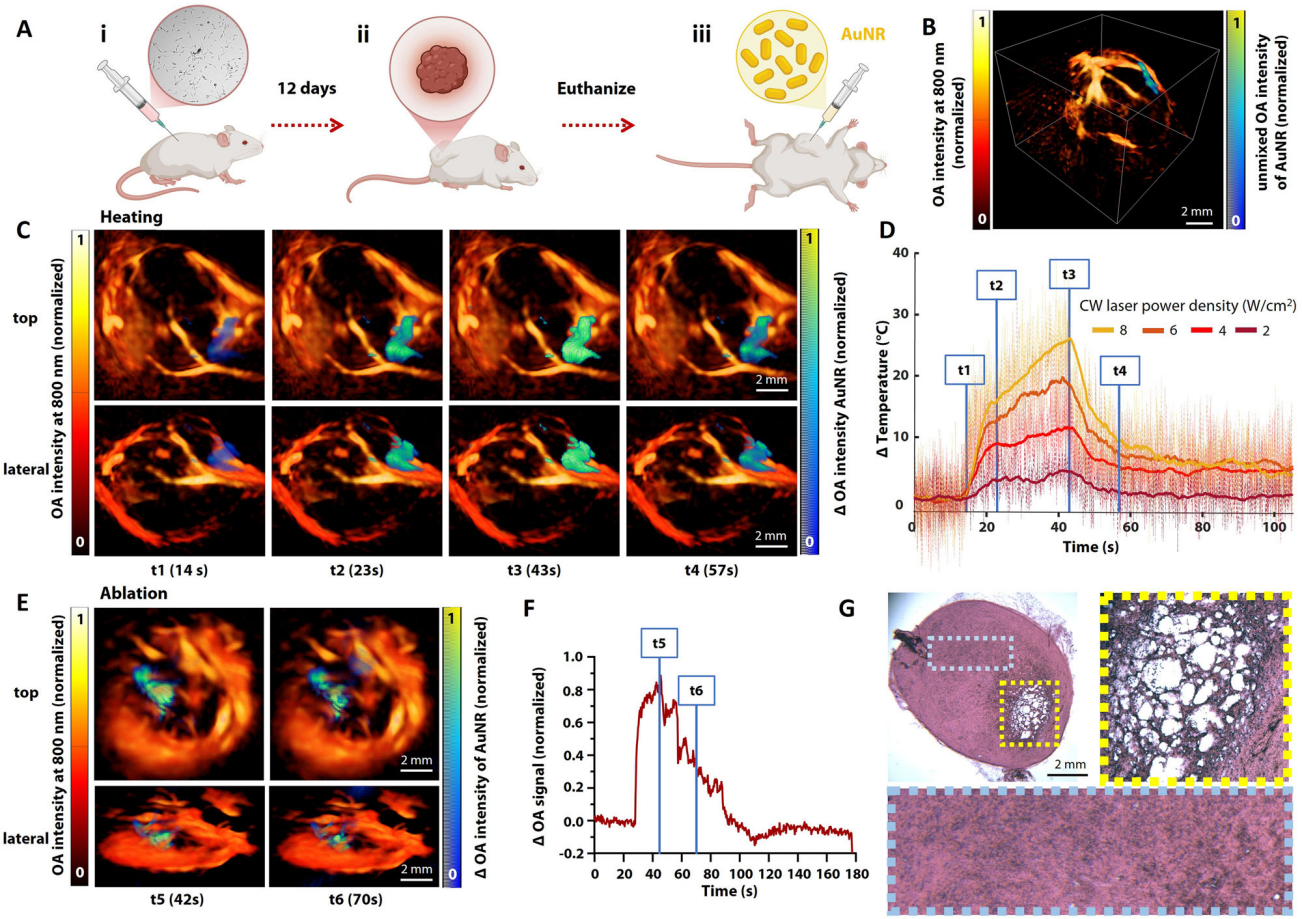
*Post mortem* imaging of tumor bearing mice. **A** (i) Mouse injected with U-87 MG glioma cells, (ii) tumor growth over the period of 12 days (iii) after euthanasia, tumor is injected with AuNRs; **B** Volumetric OA reconstruction of heat distribution during localized tumor heating; **C** Tumor heating with a CW laser (8 W/cm^2^, 1064 nm) shown at four different time points from both top and lateral perspectives; **D** Thermal profile of tumor heating for four different intensities of the CW laser and indicating the four sampled time points shown in (**C**); **E** Tumor ablation with a CW laser (16 W/cm^2^, 1064 nm) shown at two different time points from both top and lateral perspectives; **F** OA intensity profile within a region containing the AuNR, indicating the two sampled time points shown in (**E**); **G** Tumor H&E histology post localized ablation (yellow area) showing ablation zone of thermal coagulation necrosis compared to untreated tumor region in the blue area. The figure was partly created using BioRender.

OA imaging was conducted *post mortem* to evaluate localized heating and tumor ablation following photothermal therapy. U-87 MG glioma tumors were grown in mice over 12 days, after which the tumors were injected with AuNRs post-euthanasia (Fig. 3A). Spectral unmixing of volumetric OA reconstructed images revealed the spatial distribution of particles injected intratumorally during localized heating, providing insights into thermal gradients achieved during photothermal exposure (Fig. 3B and Supplementary Movie 1).

Photothermal tumor treatment was achieved under CW laser exposure at power density levels up to 8 W/cm^2^. Localized OA signal intensity increase was observed in the region where AuNR were injected, verifying selective heating of this area (Fig. 3C). The temperature increase over time, estimated from the relative increase in OA intensity, was approximately linear with laser power, as expected for temperatures below 50°C for which no tissue coagulation is produced (Fig. 3D). Tumor ablation was achieved under CW laser exposure at higher power density levels (16 W/cm^2^). Simultaneous OA imaging revealed image pattern changes in the heat-affected area, arguably corresponding to a distinct ablation zone and highlighting the progressive tumor damage in time (Fig. 3E and Supplementary Movie 2). Indeed, the relative average OA signal changes in the heated area featured an initial increase consistent with the temperature rise, followed by an irregular profile after reaching a relative increase corresponding to a temperature of approximately 50°C. These irregular changes are ascribed to tissue coagulation and cell lysis (Fig. 3F). Indeed, hematoxylin and eosin (H&E) histology confirmed localized thermal coagulation necrosis in the treated tumor area, as expected following ablation^31,32^, contrasting with the intact, untreated tumor regions (Fig. 3G). These results highlight the utility of OA imaging for accurately visualizing and quantifying the effects of thermal therapy, offering valuable real-time treatment insights for optimizing PTT ablation strategies.

## Discussion

The results presented in this work demonstrate the conceptual feasibility of real-time volumetric OA temperature monitoring of PTT assisted with AuNRs in real biological tissues. The synthesized silica-coated AuNRs were shown to exhibit photostability under short-pulsed and CW laser beams used for OA signal generation and induction of hyperthermia, respectively. The effects induced by laser-nanoparticle interactions are known to depend on the pulse duration^33,34^. Laser pulses with durations in the order of a few nanoseconds are more relevant for non-invasive diagnostics with OA or other optical imaging modalities. The use of pulse energy densities conforming with safety standards is of particular importance to ensure non-invasiveness. The coated AuNRs were shown to barely degrade after being exposed to 100 Hz pulses at 9 mJ/cm^2^ - lower than the 20 mJ/cm^2^ limit for skin exposure in the visible light range - while providing sufficient SNR so that their bio-distribution can be spectrally resolved with only a few laser pulses. It is also to be noted that higher energy densities (<100 mJ/cm^2^) are permitted for the 1064 nm excitation wavelength used in the current study^18^, for which light penetration is also maximized^35^. On the other hand, induction of hyperthermia requires using mean optical power densities beyond safety standards. CW lasers are a better alternative for this purpose as 1) they are less expensive than short-pulsed lasers with sufficient power and 2) higher temperatures are reached due to reduced melting and reshaping of AuNP^22,36^. No significant photodegradation of the synthesized coated AuNRs was observed following exposure to the CW laser beam, while sufficient temperatures were reached to result in irreversible coagulation of tissues.

The silica coating was essential to ensure photostability of the AuNRs. This is consistent with prior studies on silica coating of AuNPs of different shapes^23,37,38^, further demonstrating an increased OA signal arguably due to improved thermal interfacial conductance^39^. Other type of AuNP morphologies also demonstrated enhanced photostability with respect to standard AuNRs^40^. Noticeable changes in particle morphology were observed in the laser-exposed pristine AuNRs, also consistent with prior studies exposing AuNPs to high-intensity pulsed lasers with peak-power densities of 10^7^– 10^12^W/cm^2^^41^. A silica shell with a thickness of a few tens of nanometers was considered, previously shown to be optimal as it minimizes aggregation and electric field attenuation while ensuring photostability^42^. A key advantage of AuNRs compared to other gold nanoparticles is their flexibility to tune the LSPR peak into the NIR-II region. Indeed, most types of AuNPs have LSPR peaks in the NIR-I window below 950 nm. The growing interest in the NIR-II region mainly stems from the enhanced penetration depth, the lower background absorption, and the higher permissible exposure limit in this range. Moreover, short-pulsed Nd:YAG lasers operating at 1064 nm are widely available and suitable for efficient generation of OA signals.

Localized rapid heating around irradiated AuNPs due to photothermal transduction has been used for treating solid tumors in vivo, with both CW and nanosecond-pulsed lasers^43,44^. This approach may overcome limitations of conventional therapies like surgery, radiation, and chemotherapy, which remain the standard despite their significant drawbacks^45^. These traditional strategies often cause collateral damage to healthy tissues and are associated with undesirable side effects. Moreover, they often fail to fully eliminate tumors, leading to recurrence and treatment resistance. AuNPs can also be functionalized to target tumor microenvironments either via chemical binding or through the enhanced permeability and retention (EPR) effect^46^. Direct intratumoral injection has also been used as a more efficient strategy to deliver AuNPs into accessible tumors^47^. The *post-mortem* experiment performed in this work mimics this situation, while avoiding excessive burden to the animals according to ethical guidelines on animal experimentation. Other effects can also take place during AuNP-assisted PTT. Photomechanical effects, including cavitation, may arise when AuNPs are subjected to pulsed laser light, with the generated bubbles capable of damaging cancer cell membranes^48^. Laser irradiation of AuNPs has also been shown to lead to photochemical effects, such as the generation of reactive oxygen species, with pulsed lasers proving more effective than CW lasers^49^. The growing efforts in the development of new types of AuNPs for PTT have been fostered by recent translation of some of these particles into clinical trials^50,51^. Considering the fact that OA systems have also been certified for clinical use, the synergistic use of AuNPs and OA imaging can open new avenues in clinical treatments not limited to PTT but also including other approaches, e.g., based on high intensity focused ultrasound^52,53^.

In conclusion, the enhanced photostability of silica-coated AuNRs with absorption peaks in the NIR-II range, along with their systematic evaluation under both CW and pulsed-laser excitation, offers a robust platform for real-time, non-invasive OA temperature monitoring during PTT. By overcoming challenges related to OA signal decay caused by nanoparticle photodegradation, these AuNRs enable precise thermal mapping in deep tissue environments. The successful integration of OA imaging with temperature monitoring, validated through thermal camera readings and *post-mortem* studies, highlights the potential of this approach for improved therapeutic outcomes. This advancement not only paves the way for safer and more effective applications in cancer treatment but also opens up new possibilities for the broader use of theranostic nanomaterials in clinical settings.

## Methods

### Gold nanoparticle synthesis

Nanorod synthesis was based on a modified procedure from Vigderman and Zubarev, followed by functionalization according to Wu and Tracy^54,55^. All chemicals were purchased from Sigma-Aldrich. If not further specified, dilutions of reagents were made in Milli-Q (MQ) water (Fisher-Scientific).

To prepare the seed, a 25 mL round bottom flask equipped with a magnetic stir bar was loaded with 10 mL cetrimonium bromide (CTAB, 0.1 M) and 50 µL HAuCl_4_ (50 mM). This was heated in an oil bath set to 26°C while stirring at 400 rpm. After 10 min, 600 µL of freshly prepared NaBH_4_ (10 mM) was rapidly injected into the light yellow solution, upon which a color change to light brown was observed. Stirring continued for 30 min followed by ageing without stirring for another 30 min at 26°C. Seeds were always prepared fresh and used within 2 hours of synthesis.

For nanorod growth, a 50 mL round bottom flask equipped with a magnetic stir bar was loaded with 20 mL CTAB (0.1 M) and 200 µL (50 mM) HAuCl_4_ and heated in an oil bath set to 26°C while stirring at 400 rpm. After 10 min, 50 µL AgNO_3_ (0.1 M) was added to the dark yellow solution. After 1 min of stirring, 1 mL of freshly prepared hydroquinone (0.1 M) was rapidly injected, and the solution became transparent within 30 sec upon which 320 µL of fresh seed was rapidly injected. The mixture was then stirred for another 30 sec upon which the stir bar was removed, and the seed was allowed to grow in the dark at 26°C for 20 hours.

Excess CTAB was removed by centrifugation of the synthesized rods (2x) for 15 min at 10000 rcf at 30°C to achieve a final CTAB concentration of 10 mM and a total final volume of 10 mL. To achieve silica coating of rods, the synthesized rods were diluted with MQ water to obtain 10 mL nanorod solution at an optical density (OD) of 6 and a CTAB concentration of 1 mM. Using NaOH (5 M), the pH was adjusted to pH 9-10 while stirring at 400 rpm in an oil bath set to 30°C. Next, 330 µL of tetraethoxysilane (TEOS, 20 v/v% in MeOH) was added via syringe dropwise over a period of 5 min. Stirring was continued at 30°C and 100 rpm upon which the stir bar was removed, and the coated rods were allowed to grow undisturbed for 22 hours. Next, nanorods were centrifuged (30°C, 10000 rcf, for 15 min) and washed with MQ water (90% volume exchanged with MQ). Centrifugation was repeated a second time, upon which nanorods were resuspended in a final total volume of 5 mL at an OD of 9.5.

### Sample preparation

Two different types of experiments were conducted for characterizing the photostability of the AuNRs and the temperature estimation with OA, respectively. The first test setup consisted of a polyethylene tube placed atop the agar-filled array submerged in 1 mm agar. 10 µL of AuNRs were injected into the tubing using a syringe to minimize the sample evaporation when heated. Afterwards, the sample was collected out of the other end of the tubing, which was then replaced in between trials. The collected samples were subsequently imaged using SEM or TEM to assess the morphological alterations. The second setup consisted of placing a drop, 10 µL, onto a glass slide 20 × 20 mm placed in the center of the array, into the focal point of the pulsed and CW lasers. The focal point had been marked on the thermal camera,a allowing for consistent positioning. After the drop had been subjected to the lasers, it was collected using a pipette and secured for later SEM and TEM imaging.

### OA imaging setup

The imaging system consisted of a wavelength-tunable nanosecond laser source with a pulse repetition frequency of 100 Hz and a 512-element spherical array transducer covering an angle of 110° with 4 cm radius of curvature (1.3π solid angle). The individual elements of the array have a central frequency of 4 MHz and ~100% detection bandwidth, corresponding to near isotropic imaging resolution of ~150 µm around the geometrical center of the sphere. Acoustic coupling was ensured by filling agarose gel (1.25 g agar/100 ml Mili-Q water) between the active surface and the surface of the imaged sample. OA responses were excited with a short-pulsed (~8 ns) laser source (Innolas Laser GmbH, Krailling, Germany) guided via a custom-made fiber bundle (CeramOptec GmbH, Bonn, Germany) through a hollow cylindrical cavity in the center of the array. Neutral density filters (Thorlabs, OD 0.2 NE502B and OD 0.7 NE507B), allowing 50% and 20% of the energy to pass, respectively, were subsequently placed between the laser and the fiber bundle to attenuate and eliminate photodegradation caused by the pulsed laser. Measurements without a neutral density filter were also performed (100% transmission). For imaging, the wavelength of the tunable OA laser source was set to 1064 nm, and the optical fluence was set to approximately 9.5 mJ/cm^2^ at the surface of the imaged sample when no neutral density filter was used. All the OA signals detected by the 512 channels of the spherical array were simultaneously digitized at 40 Megasamples per second by a custom-made data acquisition (DAQ) system (Falkenstein Mikrosysteme GmbH, Taufkirchen, Germany) triggered with the Q-switch output of the laser. Additionally, the spectrum of the samples was measured by varying the wavelength in steps of 10 nm from 700 to 1100 nm to capture the full spectrum and visualize any eventual shifts.

### Laser heating

Heating of the sample was performed with a fiber-coupled continuous wave (CW) laser (Changchun New Industries Optoelectronics Technology Co., Ltd.) having a fixed wavelength of 1064 nm with a maximum power of 20 W at the output. The laser power was controlled between 10% and 40% of its maximum between trials (2 to 8 W/cm^2^ power density at the sample). The laser was turned on for up to 5 min and directed centrally onto the sample. The output of the CW laser was positioned at a distance of 40 mm from the sample, with a spot size of 1 cm^2^.

### Morphological characterization

The particle morphology of both control and exposed samples to pulsed and CW lasers for both coated and pristine batches was imaged via SEM (Hitachi SU5000) and TEM (JEOL JEM F200) to assess the degree of photodegradation. In SEM, we employed both backscattered (5.0 kV 5.5 mm S-x100k BSE-ALL) as well as secondary electron modes (10.0 kV 4.6mm L-x450k SE(L)).

### Thermal camera measurements

Temperature measurements as well as consistent positioning of the samples were ensured with a thermal camera (Optris PI, OPTPI640IO60) placed vertically above the sample. The camera was able to accurately measure temperatures of up to 100°C with an accuracy of ±2°C. This served as a reference for the temperature values estimated from the collected OA signals.

### Optoacoustic image reconstruction

Reconstruction of 3D OA images was performed with a graphics processing unit (GPU)-based implementation of the filtered back-projection algorithm, considering a voxel size of 100 x 100 x 100 mm^3^. Prior to reconstruction, the acquired time-resolved signals were band-pass filtered between 0.1 and 8 MHz, and a median filter with kernel size 3 x 3 x 3 was applied to the reconstructed images. For visualization, maximum intensity projections (MIPs) along the three Cartesian coordinates were calculated.

### OA thermometry

Temperature was estimated from OA images based on the OA signal (pressure) distribution given as:

${p}_{0}=\varGamma {\mu }_{a}\varPhi$, where *Γ* is a dimensionless Grüneisen parameter; *µ*_*a*_: optical absorption coefficient and *Φ*: the light fluence^16,29^. Additionally, the Grüneisen parameter varies in aqueous environments (*T* in °C) as follows: $\varGamma \left(T\right)=0.0043+0.0053T$, with the relative OA signal change as a function of the *T* increase Δ*T*. Then the relative signal increase to the relative temperature increase corresponds to the following formula:

1 $$\frac{\Delta {p}_{0}}{{p}_{0,0}}=\frac{0.0053\Delta T}{0.0043+0.0053{T}_{0}}$$

Furthermore, *F*_*th*_ denotes the theoretical ratio between the relative increment of the OA signal and the relative increment of temperature:

2 $${F}_{{th}}={\left(\frac{0.8113}{{T}_{0}}+1\right)}^{-1}$$

Finally, the temperature increase can be calculated as follows:

3 $$\varDelta T=\frac{{T}_{0}\varDelta {p}_{0}}{{p}_{0,0}{F}_{{th}}}$$

### Animal models

Animal experiments were performed in accordance with the Swiss Federal Act on Animal Protection and approved by the Cantonal Veterinary Office Zurich. A total of 3 athymic nude mice (Foxn1nu, Charles River Laboratories, USA, 16-18 weeks old) were used in this study. The animals were housed in individually ventilated, temperature-controlled cages under a 12-h dark/light cycle with food and water provided *ad libitum*. All animal experiments were performed *post mortem* as detailed below.

### Glioblastoma (GBM) cell lines, cell culture and subcutaneous injection

U-87 MG glioma cells were obtained from CLS Cell Lines Service GmbH, Germany (Catalog Nr: 300367). Tumor cell cultures were grown and maintained in Eagle’s Minimum Essential Medium (EMEM, Sigma Aldrich) supplemented with 10% Fetal Bovine Serum (FBS, Sigma Aldrich) in a constant temperature incubator set at 37 °C, abounding with 5% CO_2_. The culture medium was changed every 3 days. The cells were trypsinized when they reached ~90% confluency according to a logarithmic growth period. Then, a cell suspension with Phosphate-buffered saline (PBS) was prepared.

Subcutaneous GBM-bearing mice were obtained by a subcutaneous injection of 1 × 10^7^ U-87 MG cells in the regions close to the right legs of the athymic nude mice (Foxn1nu, Charles River Laboratories, Germany). Mice were euthanized and imaged 12 days after tumor cell implantation, when the average tumor size reached approximately 100 mm^3^. After the experiments, tumors were collected for histology.

### *Post mortem* experiments

The OA imaging setup included a water tank with a submerged custom-built stretcher to position the euthanized mouse. Mice were euthanized with an intraperitoneal injection of the combination of ketamine (75–100 mg/kg), xylazine (10 mg/kg) and acepromazine maleate (2–3 mg/kg) while the animal was still under isoflurane anesthesia. Following euthanasia, 20 µL of AuNR solution with the original OD of 6 and 5x diluted OD of 1.2 were injected intratumorally.

### Immunohistochemistry staining of GBM tumors

To assess AuNR-induced ablation, collected tumors were fixed overnight in 4% paraformaldehyde (PFA), then equilibrated in 15% and 30% sucrose solutions prepared in 0.1 M PBS at 4 °C. Tumors were sectioned into 30-μm-thick slices using a cryotome (CM3050S, Leica, Germany). Free-floating sections were washed with PBS, mounted onto microscope slides, and stained with H&E. Wide-field images were captured using a Zeiss microscope (Germany) to evaluate H&E staining (Abcam). Digital images were minimally processed in ImageJ to adjust brightness and contrast for visualization purposes.

### Data analysis

All acquired data was processed in MATLAB, including the OA thermometry to approximate thermodynamics based on the Grüneisen parameter, as reported previously^16^.

## Supplementary information

Supplementary Information

Supplementary Movie 1

Supplementary Movie 2

## Data Availability

The main data supporting the finding of this study are available within the main text or supplementary information. The raw datasets before image reconstruction are too large to be publicly shared, yet they are available for research purposes from the corresponding author upon request. The code that supports the findings of this study is available for research purposes from the corresponding author upon request.
