# Supplementary material for: Deep tissue optoacoustic monitoring of photothermal treatments in the NIR-II assisted with silica-coated gold nanorods
Source: Npj Imaging. 2026 Jan 28;4:4. doi: 10.1038/s44303-025-00134-7 (PMC12852917; doi:10.1038/s44303-025-00134-7)
Supplement: Supplementary file 1 — Supplementary Information [file 44303_2025_134_MOESM1_ESM.pdf]

Supplementary Information

Deep Tissue Optoacoustic Monitoring of Photothermal Treatments in the NIR-II Assisted with Silica-coated Gold Nanorods

Eva Remlova[1,2], Alexander Jessernig[3-6], Marcus Bammel[1,2], Daniil Nozdriukhin[1,2], Yi Chen[1,2], Oscar Cipolato[3-6], Xosé Luís Deán-Ben[1,2], Inge K. Herrmann[3-6], Daniel Razansky[1,2*]

[1]*Institute for Biomedical Engineering and Institute of Pharmacology and Toxicology, Faculty of Medicine, University of Zurich, Switzerland*
[2]*Institute for Biomedical Engineering, Department of Information Technology and Electrical Engineering, ETH Zurich, Switzerland*
[3]*Institute of Energy and Process Engineering, Department of Mechanical and Process Engineering ETH Zurich, Switzerland*
[4]*Laboratory for Particles-Biology Interactions, Department Materials Meet Life, Swiss Federal Laboratories for Materials Science and Technology (Empa), Lerchenfeldstrasse 5, 9014 St. Gallen, Switzerland*
[5]*Ingenuity Lab, Balgrist University Hospital, Forchstrasse 340, 8008 Zurich, Switzerland*
[6] *Faculty of Medicine, University of Zurich, Rämistrasse 71, 8006 Zurich, Switzerland*

**Corresponding author: daniel.razansky@uzh.ch (D.R.)*

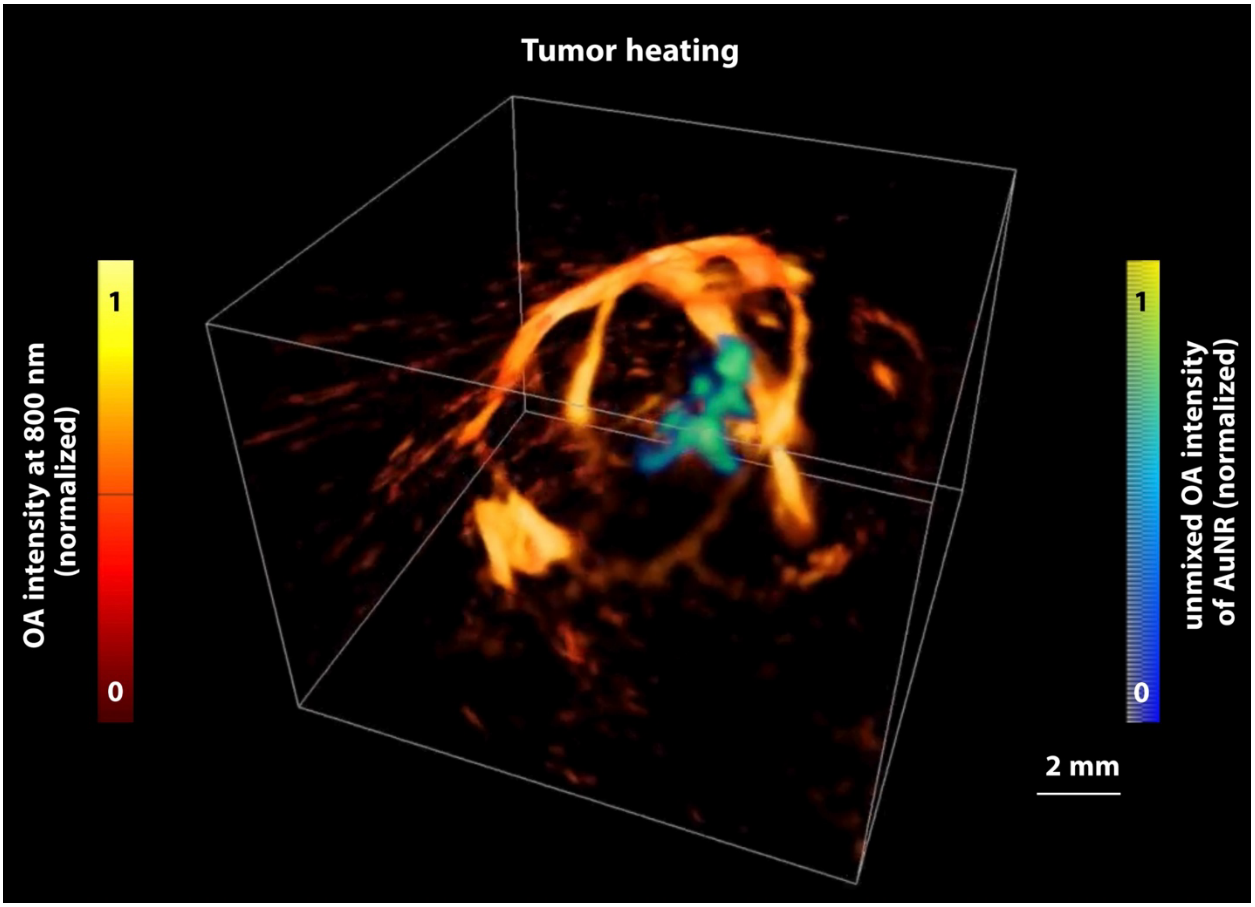

Supplementary Movie 1. Animation of volumetric OA reconstruction of heat distribution during localized tumor heating. Spectral unmixing of volumetric OA reconstructed images reveals the spatial distribution of particles injected intratumorally during localized heating, providing insights into thermal gradients achieved during photothermal exposure, in addition to Figure 3B of the main manuscript.

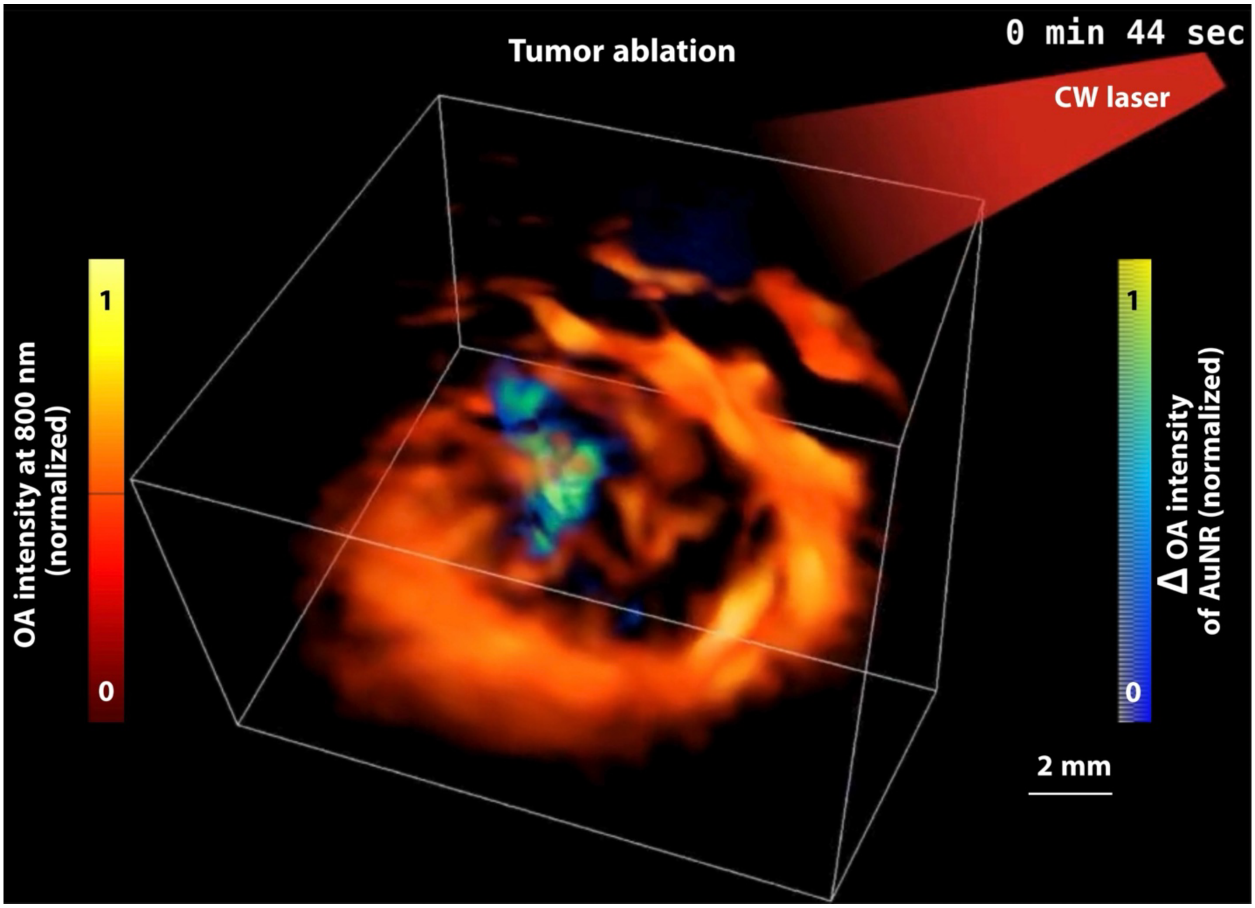

Supplementary Movie 2. Animation of the tumor ablation achieved under CW laser exposure at higher power density levels (16 W/cm^2). Simultaneous OA imaging reveals image pattern changes in the heat affected area, arguably corresponding to distinct ablation zone and highlighting the progressive tumor damage in time, complementing the respective top and lateral views presented in Figure 3E of the main manuscript.
